# Correction: Electrostatic control of regioselectivity *via* ion pairing in a Au(i)-catalyzed rearrangement

**DOI:** 10.1039/c5sc90018b

**Published:** 2015-03-25

**Authors:** Vivian M. Lau, Craig F. Gorin, Matthew W. Kanan

**Affiliations:** a Department of Chemistry , Stanford University , 337 Campus Drive , Stanford , California 94305 , USA . Email: mkanan@stanford.edu

## Abstract

Correction for ‘Electrostatic control of regioselectivity *via* ion pairing in a Au(i)-catalyzed rearrangement’ by Vivian M. Lau *et al.*, *Chem. Sci.*, 2014, **5**, 4975–4979.



## 

The first column of values in Table 3 are incorrect. Table 3 should appear as follows:

**Table 3:** Calculated dipole moments of isomeric transition states leading to products **2x** and **3x**

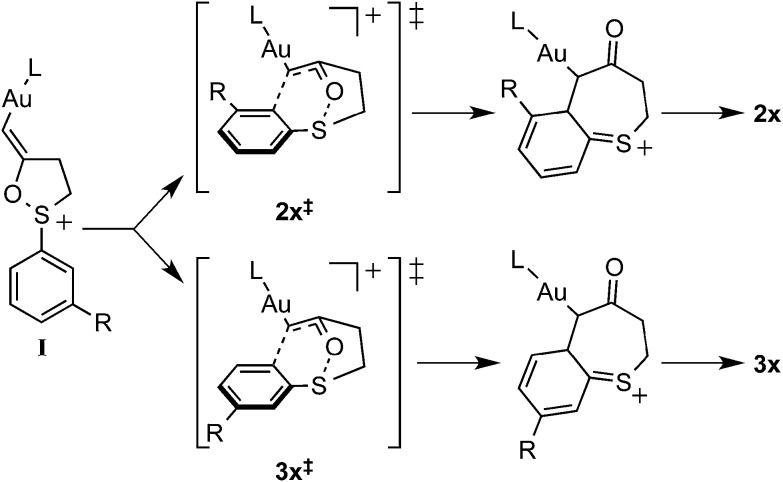				
R =	*ρ* (**2x**^**‡**^) (D)	*ρ* (**3x**^**‡**^) (D)	Δ|*ρ*| (D)	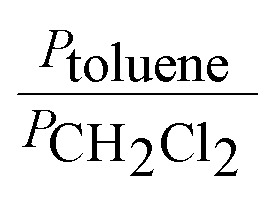 ^*a*^
Me (**1b**)	4.1	4.0	–0.1	0.9
MeO (**1c**)	4.1	4.8	0.7	1.3
F (**1e**)	2.9	5.4	2.5	3.1
Cl (**1a**)	2.6	5.9	3.3	5.0
Br (**1d**)	2.5	7.5	5.0	2.7
CF_3_ (**1f**)	2.4	9.0	6.6	6.3

^*a*^ *P*_toluene_ and *P*_CH_2_Cl_2__ are the product ratios (**3x**/**2x**) in toluene and CH_2_Cl_2_.

The Royal Society of Chemistry apologises for these errors and any consequent inconvenience to authors and readers.

